# Analysis of risk factors for papillary thyroid carcinoma and the association with thyroid function indicators

**DOI:** 10.3389/fendo.2024.1429932

**Published:** 2024-09-02

**Authors:** Jianning Liu, Zhuoying Feng, Ru Gao, Peng Liu, Fangang Meng, Lijun Fan, Lixiang Liu, Yang Du

**Affiliations:** ^1^ Center for Endemic Disease Control, Chinese Center for Disease Control and Prevention, Harbin Medical University, Harbin, Heilongjiang, China; ^2^ Key Lab of Etiology and Epidemiology, Education Bureau of Heilongjiang Province & Ministry of Health (23618504), Heilongjiang Provincial Key Lab of Trace Elements and Human Health, Harbin Medical University, Harbin, Heilongjiang, China; ^3^ Department of Physical Diagnostics, Beidahuang Industry Group General Hospital, Harbin, Heilongjiang, China

**Keywords:** PTC, TSH, iodine, BMI, ROC curve

## Abstract

**Objective:**

This study aims to analyze the relationship between papillary thyroid carcinoma (PTC) and various factors.

**Methods:**

The study involved two groups—PTC patients and non-PTC controls. We utilized binary logistic regression and Least Absolute Shrinkage and Selection Operator (Lasso) regression for variable selection and risk factor analysis. Correlation analysis was performed using Spearman’s rank correlation. The diagnostic value of thyroid stimulating hormone (TSH) levels for PTC was assessed using Receiver Operating Characteristic (ROC) curves.

**Results:**

PTC patients exhibited higher body mass index (BMI) (23.71 vs. 22.66, p<0.05) and TSH levels (3.38 vs. 1.59, p<0.05). Urinary iodine concentration (UIC) was an independent predictor of PTC (OR=1.005, p<0.05). The optimal TSH threshold for PTC diagnosis was 2.4 mIU/L [The Area Under the Curve (AUC)=67.3%, specificity=71.4%, sensitivity=70.1%]. TSH levels positively correlated with BMI (r=0.593, p<0.05) and UIC (r=0.737, p<0.05).

**Conclusions:**

UIC may be an independent predictor of PTC, and TSH levels have some diagnostic value for identifying PTC.

## Introduction

Thyroid cancer (TC) represents one of the most common malignancies within the endocrine system (1–4), arising from thyroid follicular epithelial cells or parafollicular epithelial cells (5–7). The primary types comprise papillary thyroid carcinoma (PTC), follicular thyroid carcinoma, medullary thyroid carcinoma, and anaplastic thyroid carcinoma (8, 9). Since the 1970s, the incidence of TC has steadily increased (10–13), with PTC representing up to 90% of all cases (14–20). This rise is partly attributed to the widespread use of thyroid ultrasound and fine-needle aspiration biopsy (21–23), which has led to the overdiagnosis of TC (24, 25). Compared to other types of malignant cancers, TC exhibits one of the best prognostic outcomes, with a five-year survival rate that exceeds 95% (26, 27). The primary treatment for patients with TC consists of surgical interventions, complemented by radioactive iodine therapy (28, 29). Although the treatment approaches for TC are well-established, the underlying mechanisms of its pathogenesis remain elusive (30, 31). Iodine constitutes an essential micronutrient necessary for human growth and development (3, 32), playing a crucial role in the synthesis of thyroid hormones. The relationship between thyroid diseases and iodine intake exhibits a U-shaped curve (33), indicating that both iodine deficiency and excess are closely linked to the occurrence of thyroid disorders. However, research investigating the association between iodine intake and the risk of TC continues to yield conflicting results. Following the nationwide implementation of the iodized salt program in China, the issue of inadequate iodine intake among the population was significantly mitigated. Simultaneously, high iodine intake has increasingly become a focal point of research concerning thyroid diseases (34). Urinary iodine and blood iodine constitute two crucial biomarkers that reflect the iodine intake in populations. Owing to the advantages of urinary iodine as a biomarker—its convenience, cost-effectiveness, and non-invasive nature—it is considered the preferred biomarker for assessing population iodine intake in many studies. The World Health Organization (WHO) recommends utilizing the median urinary iodine (MUI) to assess iodine intake levels (34). With the notable improvement in the national economic level, living conditions have significantly improved (35), leading to a rising incidence of overweight and obesity (36–40). In numerous studies, being overweight or obese is considered a major risk factor for several types of cancer, being associated with an increased risk of various cancers (41, 42). Although the underlying mechanisms linking overweight or obesity with the risk of TC are yet to be fully elucidated (23), some studies suggest that changes in the levels of adipocytokines may be associated with the development of TC (24). The Body Mass Index (BMI) is considered an effective composite indicator for assessing changes in weight and height (43, 44). Thyroid-stimulating hormone (TSH), secreted by the anterior pituitary gland, is crucial for maintaining the normal functioning of the thyroid gland by influencing the production of thyroid hormones. Numerous studies have demonstrated an association between serum TSH levels and the occurrence of TC, suggesting a threshold effect on the risk levels of TC (8, 10). However, some research indicates that the connection lacks significance, particularly among populations with childhood TC (45). Epidemiological studies have established that radiation-induced thyroid cancer (46, 47), particularly among individuals exposed to radiation in occupational settings and those with a history of childhood ionizing radiation (48, 49), is well-documented. In addition to this well-known high-risk factor for TC, various other factors including age, sex, vitamin D deficiency (4), family history of genetic predispositions (50), smoking, alcohol consumption (51), insulin resistance, fluoride exposure, pregnancy (52), inflammatory responses (53), and environmental pollutants (such as nitrates and heavy metals) are also considered significant contributors to the risk of TC (54). In recent years, machine learning applications have proliferated in the biomedical field. Compared to traditional statistical methods, machine learning can uncover more complex interactions among variables within datasets, thereby providing deeper insights (16). Key algorithms employed include logistic regression, Lasso regression, random forests, support vector machines, and decision trees (55). In the domain of research concerning risk factors for thyroid diseases, machine learning exhibits considerable advantages and great potential. This study aims to investigate risk factors associated with PTC using several widely-used machine learning methods.

## Materials and methods

### Study subjects

From October 2022 to February 2024, this study was conducted at Beidahuang Industry Group General Hospital in Harbin, Heilongjiang Province, China, that involved two groups of individuals. One group comprised individuals undergoing medical examinations and was found to have no thyroid nodules on thyroid ultrasound. The other group included hospitalized patients initially diagnosed with suspicious malignant thyroid nodules using thyroid ultrasound and thyroid fine needle aspiration biopsy. Their diagnoses were subsequently confirmed as PTC after a postoperative pathological examination. For both groups, we administered questionnaires, collected urine samples, measured body metrics, and gathered data on thyroid function panel, and biochemical markers. The survey questionnaire solicited information, including age, sex, height, weight, educational background, and marital status. The inclusion criteria comprised: 1) Individuals aged 18 and above; 2) Individuals with comprehensive clinical records; 3) Individuals undergoing tests for five thyroid function indicators and biochemical levels in the hospital. The exclusion criteria included: 1) Pregnant or lactating individuals; 2) Individuals suffering from severe liver or kidney failure; 3) Individuals with Type 1 diabetes; and 4) Individuals with autoimmune diseases, including autoimmune thyroid disorders. All participants provided written informed consent, and the study received approval from the Ethical Committee of Harbin Medical University.

### Physical examination, thyroid ultrasound, and thyroid fine-needle aspiration biopsy

Height and weight measurements were conducted by professional medical staff, with participants being barefoot and dressed in light clothing. The formula for calculating the BMI is the weight (in kilograms) divided by the square of the height (in meters squared, kg/m²). Prior to the thyroid ultrasound examination, patients are required to lie in a supine position. The examinations were conducted by experienced thyroid sonography specialists utilizing a 6-15 MHz transducer (LOGIQ E9), to assess the presence of thyroid nodules and to determine their sizes and quantities. Thyroid nodules are radiologically defined as discrete lesions within the thyroid gland, distinct from the surrounding thyroid tissue. The criteria for identifying the presence of thyroid nodules include lesions with a diameter of 3 mm or greater (56). For the evaluation of suspicious malignant thyroid nodules, an initial diagnosis is conducted via thyroid fine-needle aspiration (FNA) biopsy under ultrasound guidance, whereby tissue samples are extracted for pathological examination and analysis. Under ultrasound guidance, FNA should employ 22-23 gauge needles. The aspiration process should be conducted rapidly within the thyroid lesion under ultrasound guidance, and should be completed within a few seconds. The sampling procedure involves the following steps: (1) Sterilize the skin surface; (2) Locate the lesion using ultrasound guidance; (3) Insert the needle into the lesion; (4) Rapidly and repeatedly aspirate within the lesion for a few seconds to obtain cells via the needle’s cutting action; (5) Withdraw the needle and apply pressure to the puncture site to achieve hemostasis. For most ultrasound-guided fine needle aspirations, obtaining an adequate sample typically necessitates 2-3 punctures. Ultimately, the presence of PTC is determined based on the results of the postoperative pathological examination.

### Laboratory examination

Urine samples were collected from participants between 8:00 AM and 11:00 AM, without requiring fasting. Each participant’s urine sample was collected in a clean plastic tube, with a minimum volume of 5 mL, and subsequently stored at -20°C. The measurement of urinary iodine concentration (UIC) was performed using the As^3+^-Ce^4+^ catalytic spectrophotometry method, in accordance with the Chinese health standard WS/T107.1-2016. Internal quality control samples for urinary iodine level assessment were provided by the National Reference Laboratory for Iodine Deficiency Disorders (56, 57). Serum levels of TSH, free triiodothyronine (FT3), free thyroxine (FT4), thyroid peroxidase antibodies (TPOAb), and thyroglobulin antibodies (TGAb) were determined using a chemiluminescence immunoassay (specifically, the magnetic particle chemiluminescence method) provided by New Industries Biomedical Engineering Co., Ltd., Shenzhen, China. The reference ranges for these measurements were as follows: TSH, 0.3–4.5 mIU/L; FT3, 2.0–4.2 pg/mL; FT4, 0.8–1.72 ng/dL; TGAb, 0–100 IU/mL; TPOAb, 0.38–16 IU/mL. Serum levels of potassium (K), sodium (Na), chloride (Cl), calcium (Ca), and uric acid (UA) were assessed using a fully automated Beckman AU 5800 biochemical analyzer, manufactured by Beckman Coulter, China.

### Statistical analysis

Data collection was conducted using Microsoft Office Excel 2019, while statistical analyses were carried out using IBM SPSS Statistics Version 26. The Kolmogorov-Smirnov test was utilized to evaluate the normality of the data. Continuous variables with a normal distribution were characterized as mean ± standard deviation (mean ± SD) and analyzed using independent samples t-tests. For continuous variables not normally-distributed, the 25th and 75th percentiles were utilized for characterization, and analyses were conducted using the Mann-Whitney U test. Categorical variables were depicted as counts or percentages and evaluated using the chi-square test or Fisher’s exact test. Spearman’s rank correlation analysis was utilized to investigate the relationship between TSH levels and other factors. Binary logistic regression analysis was conducted to assess risk factors for PTC, with odds ratios (OR) and 95% confidence intervals (95% CI) being calculated to elucidate their associations with PTC. The Least Absolute Shrinkage and Selection Operator (Lasso) regression analysis represents a method of shrinkage and variable selection for linear regression models. Lasso regression analysis imposes constraints on model parameters, resulting in some regression coefficients shrinking to zero. Variables whose coefficients shrink to zero during this process are excluded from the model, whereas those with non-zero coefficients are identified as strongly correlated with the response variable. This method enhances model performance, not by excluding more independent variables beyond a certain threshold, but by precisely identifying the most significant predictors. The Lambda.1se is selected to derive models that exhibit excellent performance and incorporate a minimal number of independent variables. The Lasso method is utilized to analyze the data and identify the optimal predictors of current risk factors. Lasso regression analyses were conducted using the “glmnet” package in R version 4.2.2 to screen for risk factors associated with PTC; furthermore, plots of Lasso coefficient paths and Lasso regularization paths were generated. The “pROC” package was employed to generate Receiver Operating Characteristic (ROC) curve plots. Bubble charts were generated employing the “ggplot2” package. A p-value of less than 0.05 was deemed statistically significant.

## Results

### Study population and general information

**Figure 1:**
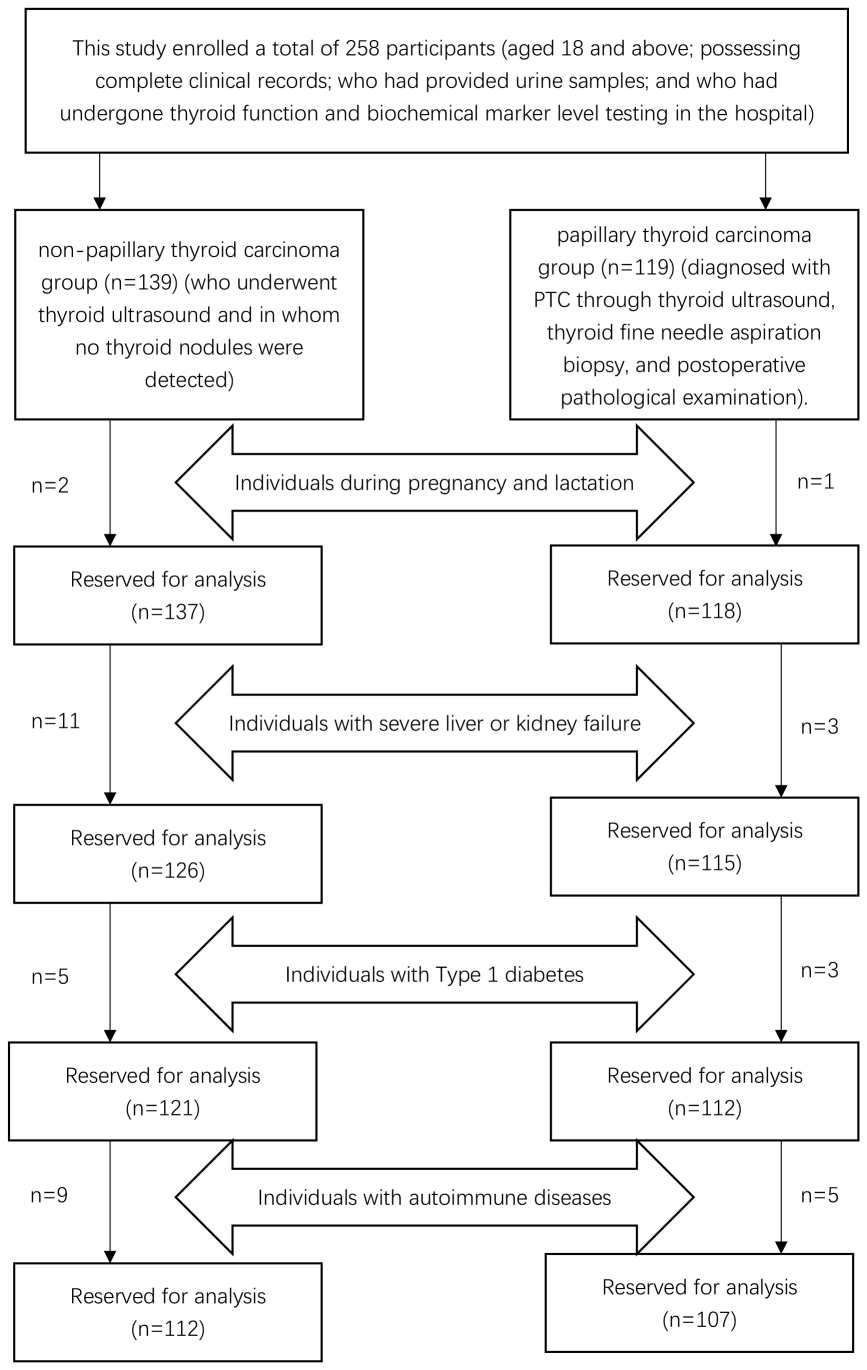
Flowchart of the participants’ screening process.

This study enrolled a total of 258 participants (aged 18 and above; possessing complete clinical records; who had provided urine samples; and who had undergone thyroid function and biochemical marker level testing in the hospital): the first group of individuals (who underwent thyroid ultrasound and in whom no thyroid nodules were detected) was classified as the non-papillary thyroid carcinoma group (n=139), and the second group of inpatients (diagnosed with PTC through thyroid ultrasound, thyroid fine needle aspiration biopsy, and postoperative pathological examination) was classified as the papillary thyroid carcinoma group (n=119). In the non-papillary thyroid carcinoma group, in accordance with the exclusion criteria, two individuals who were pregnant or lactating, eleven with severe hepatic or renal dysfunction, five with Type 1 diabetes, and nine with autoimmune diseases were excluded. Ultimately, a total of 112 participants were retained in the non-papillary thyroid carcinoma group. In the papillary thyroid carcinoma group, in accordance with the exclusion criteria, one pregnant or lactating inpatient, three inpatients with severe hepatic or renal dysfunction, three inpatients with Type 1 diabetes, and five inpatients with autoimmune diseases were excluded. Ultimately, a total of 107 participants were retained in the papillary thyroid carcinoma group, as depicted in **Figure 1**. Age, sex, BMI, educational background, and marital status for both groups are detailed in **Table 1**. No statistically significant differences were observed between the groups in terms of age (54.99 vs. 57.08, t=1.52, p=0.13), sex (χ²=0.12, p=0.76), and education levels (χ²=2.28, p=0.52), with the majority having completed middle or high school education and a minority possessing either elementary education or higher academic degrees. However, significant statistical differences were noted in BMI (23.71 vs. 22.66, t=-3.36, p<0.05) and marital status (χ²=11.12, p<0.05). The majority of participants in both groups were married, with unmarried, widowed, and divorced individuals constituting a smaller proportion.

**Table 1:** Basic information for papillary thyroid carcinoma group and non-papillary thyroid carcinoma group.

Variables	Thyroid Cancer (-)	Thyroid Cancer (+)	t (χ^2^)	P
Age (year)	57.08 ± 9.55	54.99 ± 10.79	1.52	0.13
BMI (kg/m²)	22.66 ± 2.21	23.71 ± 2.38	-3.36	<0.05
Sex			0.12	0.76
Male	27 (24%)	28 (26%)		
Female	85 (76%)	79 (74%)		
Marital Status			11.12	<0.05
Unmarried	0 (0%)	3 (3%)		
Married	98 (88%)	87 (81%)		
Widowed	14 (12%)	10 (9%)		
Divorced	0 (0%)	7 (7%)		
Education Level			2.28	0.52
Primary school and below	18 (16%)	22 (21%)		
Junior high school	56 (50%)	43 (40%)		
Senior high school	30 (27%)	32 (30%)		
College and above	8 (7%)	10 (9%)		

Thyroid Cancer (-), non-papillary thyroid carcinoma group; Thyroid Cancer (+), papillary thyroid carcinoma group; BMI, body mass index.

### Comparison of thyroid function indicators

**Figure 2:**
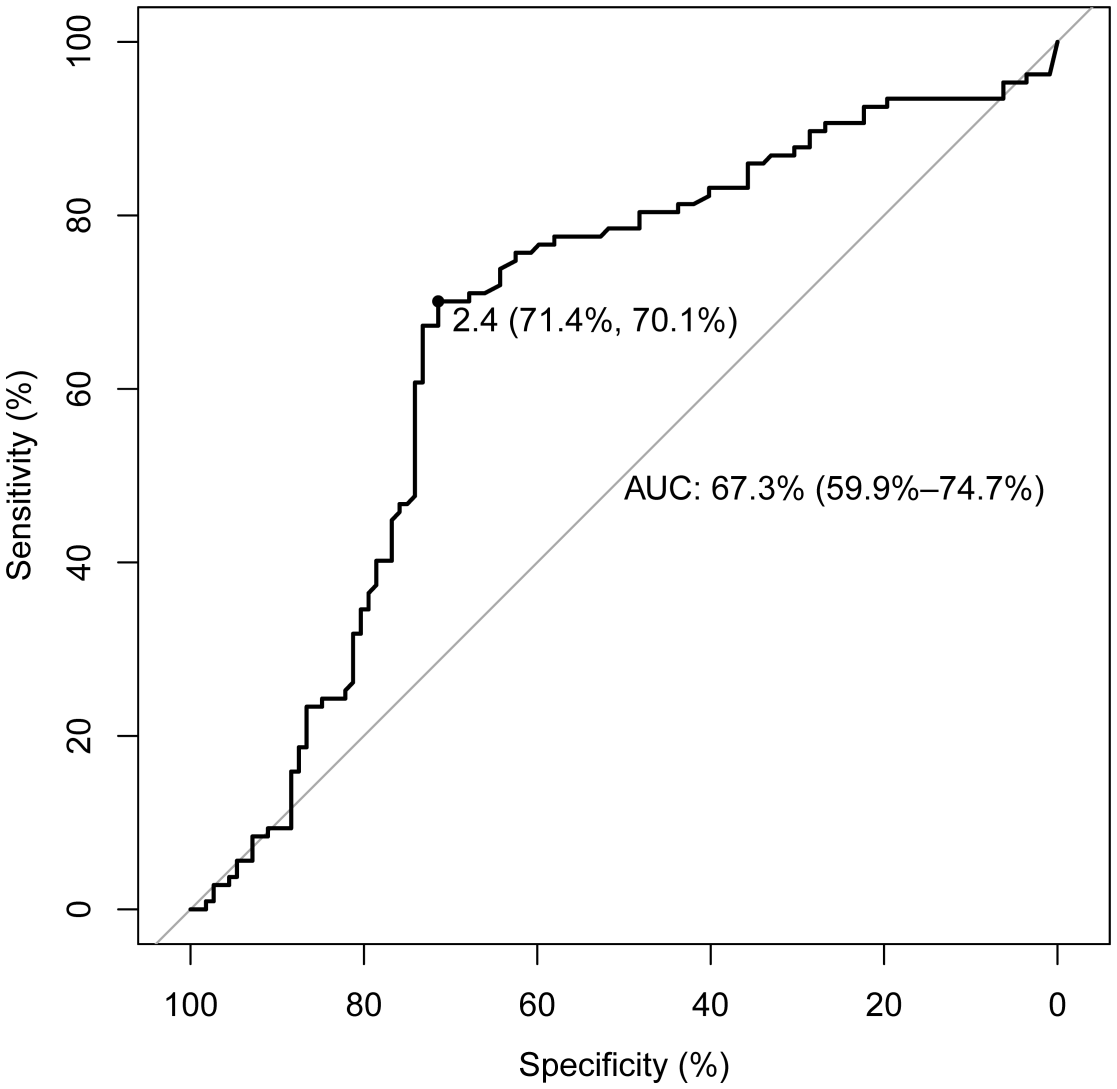
ROC curve for determining the TSH threshold in papillary thyroid carcinoma patients. The threshold for TSH concentration is set at 2.4 mIU/L, with a sensitivity of 70.1% and a specificity of 71.4%. The AUC is 67.3% (95% CI: 59.9%-74.7%).

As indicated in **Table 2**, a statistically significant difference exists between the two groups in terms of TSH levels (3.38 vs. 1.59, Z=-4.43, p<0.05). No statistical differences were noted in FT3 (3.04 vs. 3.05, Z=-0.54, p=0.59), FT4 (1.26 vs. 1.28, Z=-0.72, p=0.48), TGAb (14.7 vs. 11.95, Z=-0.26, p=0.80), or TPOAb (2.32 vs. 2.39, Z=-0.26, p=0.79). In this study, with PTC status designated as the dependent variable and TSH as the independent variable, the ROC curve analysis was utilized to identify the optimal TSH threshold for distinguishing between the two groups. The identified optimal TSH threshold was established at 2.4 mIU/L, exhibiting a specificity of 71.4% and a sensitivity of 70.1%. The area under the curve (AUC) was calculated to be 67.3% (95% CI: 59.9%-74.7%), as illustrated in **Figure 2**.

**Table 2:** Comparison of thyroid function indicators between papillary thyroid carcinoma group and non-papillary thyroid carcinoma group group.

Variables	Thyroid Cancer (-)	Thyroid Cancer (+)	Z	P
TSH (mIU/L)	1.59 (0.92,3.46)	3.38 (2.1,4.45)	-4.43	<0.05
FT3 (pg/mL)	3.05 (2.78,3.34)	3.04 (2.82,3.35)	-0.54	0.59
FT4 (ng/dL)	1.28 (1.14,1.41)	1.26 (1.13,1.4)	-0.72	0.48
TGAb (IU/mL)	11.95 (5.28,24.15)	14.7 (5,25.6)	-0.26	0.8
TPOAb (IU/mL)	2.39 (1.26,4.67)	2.32 (1.26,4.48)	-0.26	0.79

Thyroid Cancer (-), non-papillary thyroid carcinoma group; Thyroid Cancer (+), papillary thyroid carcinoma group; TSH, thyroid stimulating hormone; FT3, free triiodothyronine; FT4, free thyroxine; TGAb, thyroglobulin antibodies; TPOAb, thyroid peroxidase antibodies.

### Comparison of serum ions, uric acid, and urinary iodine concentrations

As demonstrated in **Table 3**, a statistically significant difference was observed between the two groups in terms of UIC (226.45 vs. 121.23, Z=-4.99, p<0.05). However, no significant differences were detected in K (3.95 vs. 4.00, t=0.99, p=0.32), Na (141.72 vs. 141.74, t=0.07, p=0.95), Cl (106.01 vs. 105.76, t=-0.51, p=0.62), Ca (2.30 vs. 2.28, Z=-0.87, p=0.39), or UA (300 vs. 292, Z=-0.77, p=0.44).

**Table 3:** Comparison of serum ions, uric acid, and urinary iodine concentrations between the papillary thyroid carcinoma group and the non-papillary thyroid carcinoma group.

Variables	Thyroid Cancer (-)	Thyroid Cancer (+)	t (Z)	P
K (mmol/L)	4.00 ± 0.42	3.95 ± 0.37	0.99	0.32
Na (mmol/L)	141.74 ± 2.33	141.72 ± 2.23	0.07	0.95
Cl (mmol/L)	105.76 ± 2.97	106.01 ± 4.31	-0.51	0.62
Ca (mmol/L)	2.28 ± 0.11	2.30 ± 0.13	-0.87	0.39
UA (umol/L)	292 (240,343)	300 (252,343)	-0.77	0.44
UIC (mg/L)	121.23 (98.76,233.72)	226.45 (155.58,293.68)	-4.99	<0.05

Thyroid Cancer (-), non-papillary thyroid carcinoma group; Thyroid Cancer (+), papillary thyroid carcinoma group; K, serum potassium; Na, serum sodium; Cl, serum chloride; Ca, serum calcium; UA, serum uric acid; UIC, urinary iodine concentration.

### Independent predictive factors for PTC

**Figure 3:**
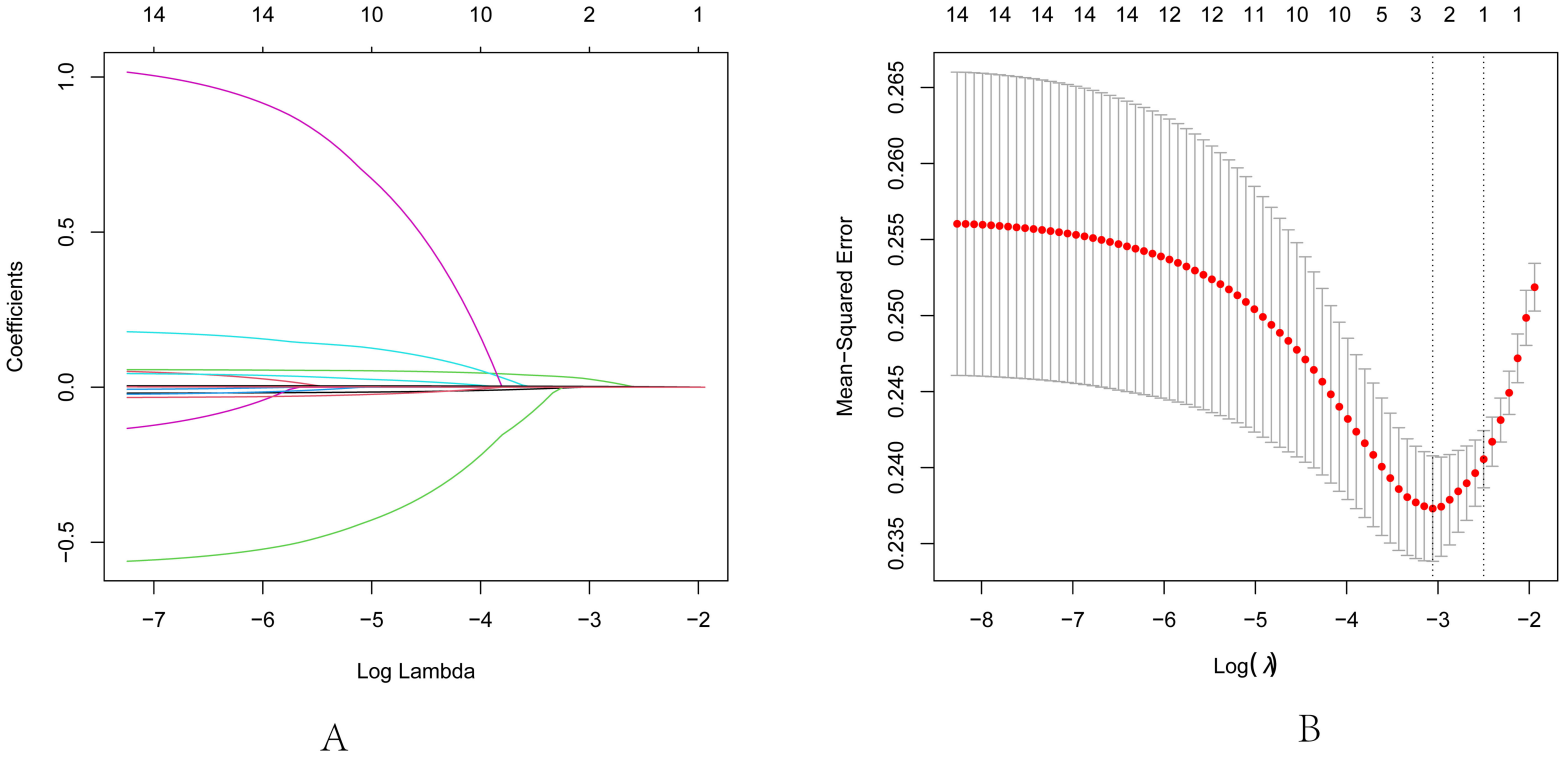
Lasso regression analysis for feature selection. **(A)** Lasso coefficient path diagram: The x-axis represents log-lambda, reflecting the degree of regularization. It shows that as the lambda value increases, the coefficient values reduce gradually to zero, and the number of retained variables decreases. **(B)** Lasso regularization path diagram: The x-axis is log (lambda); the upper axis denotes the number of non-zero coefficients, while the left y-axis represents the Mean-Squared Error (MSE). This graph demonstrates the variation in MSE with different lambda values and the confidence interval of MSE ± one standard deviation. The two vertical lines in the diagram represent the two results selected by the algorithm. The left dashed line is lambda.min, where the lambda value minimizes the MSE, and the right dashed line is lambda.1se, where the lambda value maintains the MSE within one standard error of the minimum MSE, thereby reducing the complexity of the model.

In our study, PTC was designated as the dependent variable, and the initial binary logistic regression model was constructed with K, Na, Cl, Ca, UA, and UIC serving as independent variables. The analysis revealed a significant positive association between UIC (OR=1.005, 95% CI: 1.002-1.007, p<0.05) and the prevalence of PTC, while no significant associations emerged for K, Na, Cl, Ca, and UA in relation to PTC. Upon further incorporating age, sex, and BMI as covariates, a second binary logistic regression model was developed. In this model, although BMI exhibited significance in univariate analysis (OR=1.047, 95% CI: 0.907-1.21, p=0.53), it did not retain its statistical significance. However, UIC (OR=1.004, 95% CI: 1.001-1.007, p<0.05) continued to be significantly associated with an increased risk of PTC. Finally, upon integrating five thyroid function indicators into the third binary logistic regression model, TSH, despite its significance in univariate analysis (OR=0.991, 95% CI: 0.894-1.1, p=0.869), did not exhibit statistical significance in this iteration. Yet, the association between UIC (OR=1.005, 95% CI: 1.001-1.008, p<0.05) and the risk of PTC continued to demonstrate positive significance, as illustrated in **Table 4**. To further validate the results of the third binary logistic regression model, PTC was employed as the dependent variable and all independent variables from the third model were incorporated into a Lasso regression model for variable selection. As illustrated in **Figure 3A**, the increase in the lambda value is observed to coincide with a gradual decrease in the coefficient values until they reach zero. Correspondingly, the horizontal axis demonstrates that there is a reduction in the number of variables retained as the lambda value increases. As depicted in **Figure 3B**, the left dashed line (lambda.min=0.047) identifies the model minimizing the Mean-Squared Error (MSE), whereas the right dashed line (lambda.1se=0.082) signifies the model that lies within one standard error of the minimum MSE, albeit with reduced complexity. Pursuing the model at the lambda.min value, BMI and UIC remained as independent variables. However, this study selected the model at the lambda.1se value as the optimal model, with UIC emerging as the sole variable, as shown in **Table 5**.

**Table 4:** Establishment of a binary logistic regression model for papillary thyroid carcinoma.

Model 1					Model 2					Model 3				
		OR (95% CI)					OR (95% CI)					OR (95% CI)		
Variables	OR	Lower limit	Upper limit	P	Variables	OR	Lower limit	Upper limit	P	Variables	OR	Lower limit	Upper limit	P
K (mmol/L)	0.55	0.256	1.18	0.125	Age	0.982	0.955	1.011	0.224	Age	0.981	0.952	1.01	0.197
Na (mmol/L)	0.967	0.837	1.117	0.651	BMI (kg/m²)	1.047	0.907	1.21	0.53	BMI (kg/m²)	1.059	0.913	1.228	0.448
Cl (mmol/L)	1.045	0.95	1.15	0.369	Sex (Female v.s. Male)	1.035	0.534	2.008	0.919	Sex(Female v.s. Male)	1.063	0.544	2.077	0.859
Ca (mmol/L)	3.89	0.3	50.466	0.299	K (mmol/L)	0.569	0.262	1.234	0.153	K (mmol/L)	0.561	0.254	1.238	0.152
UA (umol/L)	1.001	0.997	1.005	0.54	Na (mmol/L)	0.957	0.827	1.108	0.56	Na (mmol/L)	0.975	0.838	1.133	0.738
UIC (mg/L)	1.005	1.002	1.007	<0.05	Cl (mmol/L)	1.051	0.954	1.159	0.314	Cl (mmol/L)	1.048	0.948	1.158	0.364
					Ca (mmol/L)	3.41	0.26	44.791	0.351	Ca (mmol/L)	2.879	0.211	39.311	0.428
					UA (umol/L)	1.001	0.997	1.005	0.538	UA (umol/L)	1.001	0.997	1.005	0.526
					UIC (mg/L)	1.004	1.001	1.007	<0.05	UIC (mg/L)	1.005	1.001	1.008	<0.05
										TSH (mIU/L)	0.991	0.894	1.1	0.869
										FT3 (pg/mL)	1.207	0.827	1.763	0.33
										FT4 (ng/dL)	0.844	0.174	4.093	0.833
										TGAb (IU/mL)	1.005	0.988	1.022	0.598
										TPOAb (IU/mL)	0.966	0.886	1.054	0.438

BMI, body mass index; K, serum potassium; Na, serum sodium; Cl, serum chloride; Ca, serum calcium; UA, serum uric acid; UIC, urinary iodine concentration; TSH, thyroid stimulating hormone; FT3, free triiodothyronine; FT4, free thyroxine; TGAb, thyroglobulin antibodies; TPOAb, thyroid peroxidase antibodies.

**Table 5:** Coefficient table for predictor selection via lasso regression.

Variables	Coef (lambda.min=0.047)	Coef (lambda.1se=0.082)
Age	.	.
BMI	0.0087	.
Sex(Female v.s. Male)		.
K (mmol/L)	.	.
Na (mmol/L)	.	.
Cl (mmol/L)	.	.
Ca (mmol/L)	.	.
UA (umol/L)	.	.
UIC (mg/L)	0.0005	0.0003
TSH (mIU/L)	.	.
FT3 (pg/mL)	.	.
FT4 (ng/dL)	.	.
TGAb (IU/mL)	.	.
TPOAb (IU/mL)	.	.

BMI, body mass index; K, serum potassium; Na, serum sodium; Cl, serum chloride; Ca, serum calcium; UA, serum uric acid; UIC, urinary iodine concentration; TSH, thyroid stimulating hormone; FT3, free triiodothyronine; FT4, free thyroxine; TGAb, thyroglobulin antibodies; TPOAb, thyroid peroxidase antibodies.

### The correlation analysis of TSH

**Figure 4:**
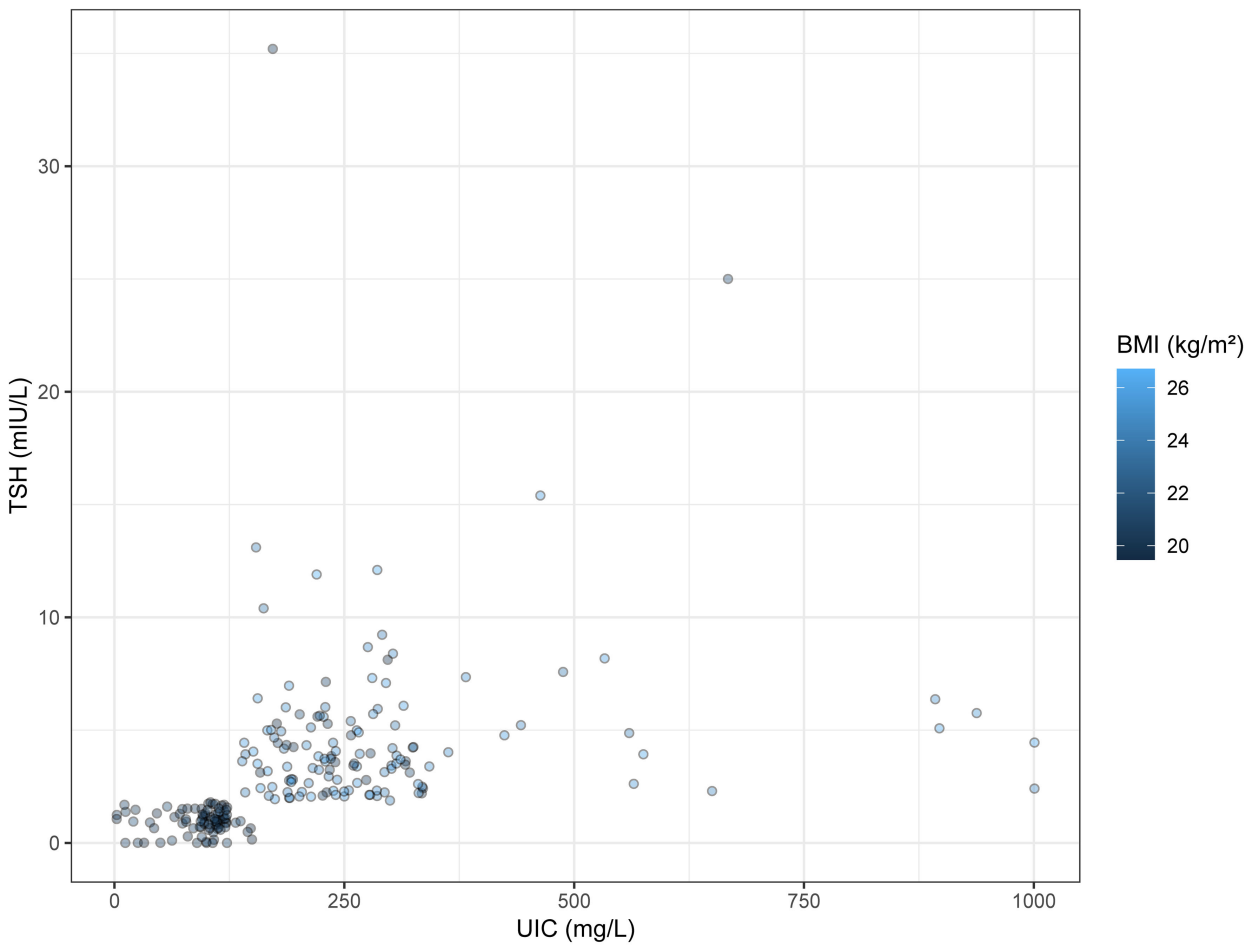
Bubble Chart. The x-axis represents UIC, and the y-axis indicates TSH concentration. The colors of the circles denote BMI values.

Based on the outcomes of our previous analysis, Spearman’s rank correlation was employed to further scrutinize the correlation between TSH and other independent variables. The findings indicated a positive correlation between TSH and both BMI (r=0.593, p<0.05) and UIC (r=0.737, p<0.05). With an increase in UIC, a corresponding rise in TSH levels is observed; conversely, an increment in TSH levels is associated with a tendency for the circle color to become lighter, as demonstrated in **Table 6** and **Figure 4**.

**Table 6:** Spearman rank correlation analysis for TSH.

Variables	TSH (mIU/L)	
	r	p
Age	0.004	0.949
BMI	0.593	<0.05
Sex(Female v.s. Male)	-0.096	0.156
K (mmol/L)	-0.018	0.796
Na (mmol/L)	-0.089	0.191
Cl (mmol/L)	-0.031	0.645
Ca (mmol/L)	0.041	0.549
UA (umol/L)	-0.062	0.361
UIC (mg/L)	0.737	<0.05

BMI, body mass index; K, serum potassium; Na, serum sodium; Cl, serum chloride; Ca, serum calcium; UA, serum uric acid; UIC, urinary iodine concentration; TSH, thyroid stimulating hormone; r, correlation coefficient.

## Discussion

TC represents the most common malignant tumor within the endocrine system (1, 53), with surgery as the primary treatment modality for affected patients. The underlying mechanisms of TC development remain elusive (13, 54), rendering research into the risk factors of TC critically important for its prevention. Numerous studies have demonstrated that estrogen promotes mitosis in thyroid cancer cells (36, 43), with estrogen receptors highly expressed in these cancers (35). Upon binding with these receptors, there is an increased probability of proliferation in thyroid cancer cells (25, 41). However, the biological mechanisms through which sex hormones might facilitate the progression of TC remain undetermined and warrant further evaluation. In our study, we observed no significant differences in sex or age between the papillary thyroid carcinoma group and the non-papillary thyroid carcinoma group.

Large-scale epidemiological studies utilizing the Global Burden of Disease (GBD) public database have identified a high BMI as a significant risk factor for TC (13). Extensive research exploring the biological mechanisms that link overweight or obesity to an increased risk of TC is currently being undertaken by numerous medical research institutions. These studies primarily focus on a range of biological explanations. Firstly, insulin resistance may play a prominent role in the onset and progression of TC (22, 30). Insulin and insulin-like growth factors (IGFs) share receptor homology, thereby enabling insulin to influence the synthesis and biological activity of insulin-like growth factor-1 (IGF-1) (30, 37, 48). While insulin exerts an inducing effect on tumor growth, numerous physiological activities, including the regulation of human aging processes, are mediated through IGF-1 (35). Numerous tissues are capable of secreting IGFs, which primarily act in an autocrine or paracrine manner to stimulate tissue proliferation and differentiation (39, 42). Specifically, IGF-1 is closely associated with TSH-mediated thyroid cell proliferation and is considered a critical pathway in regulating thyroid gene expression (35, 42), as well as the proliferation and differentiation of thyroid cells, through the insulin/IGF-1 signaling pathway (39, 43, 48). Additionally, a majority of cancer tissues express insulin-like growth factor receptors. A study found that the expression of insulin-like growth factor-1 receptor (IGF-1R) protein and mRNA in thyroid tissues of patients with PTC was markedly higher compared to that in a healthy control group (30). Furthermore, individuals with elevated circulating levels of IGF-1 exhibit an increased risk for certain types of cancer (24). This phenomenon could be attributed to the oncogenic effects of elevated IGF-1 levels (43), which are known to promote cell mitosis and inhibit apoptosis, processes that are crucial in both cell proliferation and the inhibition of cell apoptosis. Being overweight or obese is a significant risk factor for metabolic syndrome, a condition characterized by chronically elevated levels of circulating insulin (30). The prevalence of insulin resistance is particularly high among individuals who are overweight or obese. This association may partly explain the increased risk of TC among individuals who are overweight or obese (24). Furthermore, adipose tissue secretes adipokines such as leptin, adiponectin, and prostaglandins (36, 40), which under normal physiological conditions act primarily locally in adipose tissue or affect distant target organs via the bloodstream, regulating their growth, metabolism, and tissue restructuring. However, under pathological conditions, the synthesis and secretion of adipokines become dysregulated, thereby stimulating the hypothalamic-pituitary-thyroid axis and leading to increased TSH secretion (8, 28), which promotes cell proliferation (35, 44). A perspective suggests that the binding of TSH to its receptors on adipocytes stimulates the production of interleukin-16 (IL-16) (8, 39), which mediates leptin secretion (28). Leptin may disrupt the negative feedback regulation of thyroid hormones. Additionally, leptin can directly act on thyrotropin-releasing hormone (TRH) neurons through its receptors on thyroid cells, thereby influencing TRH expression (39). As body fat increases, so too does the level of leptin secretion. In this study, univariate analysis revealed that the BMI of the papillary thyroid carcinoma group was significantly higher than that of the non-papillary thyroid carcinoma group. Furthermore, a positive correlation was observed between TSH levels and BMI, with TSH levels increasing concomitantly as BMI increased. Another study on the development of TC in relation to Bisphenol A (BPA) exposure found an interaction between BPA exposure and excessive adipose tissue, promoting the occurrence of TC. BPA in the environment tends to be stored in adipose tissue, and continuous low-level BPA exposure exacerbates chronic inflammation in these tissues. After adjusting for the study model, it was determined that metabolic syndrome is a necessary condition for BPA to promote TC. BPA may further aggravate insulin resistance, thereby influencing the incidence of TC (58). However, many studies have confirmed the increased risk of TC associated with metabolic syndrome, which is a controllable risk factor. Overweight and obesity are significant components of metabolic syndrome. Guiding the public to focus on weight management and maintaining a reasonable weight is crucial for reducing the risk of TC (59).

Iodine is an essential trace element (15, 34), crucial for the healthy development of children and pregnant women. Iodine deficiency can severely impair children’s brain development and cognitive functions. Furthermore, iodine is a vital component for the synthesis of thyroid hormones (8, 33), which are essential for promoting the metabolism of sugars and lipids, facilitating growth and development, and maintaining metabolic homeostasis within the body. In the past, severe iodine deficiency was widespread across most regions of China, where dietary iodine was predominantly obtained from fish and seafood (33). In recent years, the implementation of iodized salt programs has significantly improved the iodine nutrition status in regions previously deficient in iodine. This has subsequently led to an excess of iodine in many areas. Epidemiological studies have found that an increase in iodine intake is positively correlated with the risk of PTC (35), and that the ratio of PTC to follicular thyroid cancer is correlated with iodine intake levels. In certain iodine-rich areas of China, research has revealed that the mutation rate of the BRAF gene exhibits a positive correlation with the iodine concentration in drinking water (15). This data suggests that high iodine intake could potentially trigger BRAF mutations (34), which may indirectly lead to the development of PTC (40). Another epidemiological study indicates that after iodine is ingested from external sources, it is subsequently transported into thyroid cells by the sodium-iodide symporter (NIS) (8). Iodine, hydrogen peroxide, and thyroglobulin (Tg) are then oxidized by thyroid peroxidase (TPO) to form iodinated thyroglobulin. Prolonged high iodine intake could accelerate this process, potentially leading to a shortage of hydrogen peroxide. This imbalance could trigger thyroid inflammation or the development of tumors. In a laboratory study, it was observed that iodine upregulates MAPK1 and inhibits miR-422a (34), thereby potentially stimulating the progression of TC. In this study, urine samples were collected from participants to measure urinary iodine concentrations (15). Typically, more than 80% of daily iodine intake is excreted in urine, rendering urinary iodine an effective biomarker of population iodine intake (34). In this research, urinary iodine levels emerged as an independent predictive factor for PTC. Higher urinary iodine concentrations might exhibit a positive correlation with the risk of developing PTC. Furthermore, analyses were conducted on K, Na, Cl, and Ca levels, yet no significant correlation was observed between these elements and the incidence of PTC.

TSH is recognized as a critical hormone secreted by the anterior pituitary gland, playing an integral role in the regulation of thyroid function. This encompasses the modulation of thyroid cell proliferation, in addition to the secretion and synthesis of thyroid hormones and the regulation of the thyroid’s blood supply (39). Pituitary disorders may directly impair the synthesis and release of TSH, while hypothalamic diseases can affect the secretion of thyrotropin-releasing hormone (TRH), thereby indirectly modulating TSH secretion (8). TSH modulates the secretion of thyroid hormones and regulates basal energy expenditure, thus potentially contributing to the etiology of TC. TSH plays a role in the mitotic activities of the thyroid gland (22), and a positive correlation has been observed between TSH levels and the risk of TC (36). Prolonged exposure to elevated levels of TSH may stimulate thyroid cell proliferation and elevate the risk of malignant mutations in these cells (14, 31, 45), potentially precipitating the onset of TC (6, 43). An additional biological mechanism proposed involves TSH potentially influencing the downregulation of p53 protein expression, which may affect the progression of TC. The specific biological mechanisms warrant further investigation (8). The relationship between TSH and TC is multifaceted. Determining whether TSH acts as a trigger for the onset of TC or as a promoter of its progression underscores the importance of further research into the connection between TSH and TC (10). In this investigation, the levels of TSH were observed to be significantly higher in the papillary thyroid carcinoma group than in the non-papillary thyroid carcinoma group. The ROC curve was utilized to evaluate the diagnostic value of TSH in PTC. A threshold concentration of TSH was identified at 2.4 mIU/L, with an area under the curve (AUC) of 67.3%, and both sensitivity and specificity exceeded 70%. Moreover, Spearman’s rank correlation analysis was employed to assess the relationship between TSH and other indicators. It was found that BMI and UIC were positively correlated with TSH, suggesting that TSH levels could be influenced by these factors. Uric acid (UA), the end product of purine metabolism, acts as an antioxidant (60). High levels of UA have been associated with various diseases. Epidemiological studies have suggested that elevated UA levels may constitute a risk factor for the development of thyroid nodules (61). However, there have been no prior studies addressing the relationship between UA and PTC. In this investigation, the connection between UA and PTC was analyzed. A clear link between UA levels and the presence of PTC was not found. Finally, the significance of our study lies in its ability to provide direction for exploring the etiology of PTC through statistical analysis of potential risk factors. Furthermore, regarding disease prevention, the public should consider their own conditions. High-risk individuals, in particular, should monitor changes in their indicators and undergo timely health check-ups.

This study has several limitations. Firstly, our study population, primarily derived from a hospital setting, posed challenges in avoiding selection bias in participant selection, diagnosis of target groups, and study implementation. Future research should aim to be conducted across multiple hospitals to consider diverse risk factors, foster collective intelligence, and enhance experimental design quality. Secondly, Future research should expand the sample size and include different geographic regions to validate the generalizability of the current results. Lastly, although 24-hour urinary iodine excretion is an effective indicator of overall iodine levels, its reliance on urine samples makes it susceptible to external influences such as diet. Despite these limitations, this research continues to provide valuable directions and insights for studies on PTC.

## Conclusions

UIC serves as an independent predictive factor for the occurrence or progression of PTC. TSH possesses significant diagnostic value for PTC, with a defined diagnostic threshold. A close relationship has been observed between TSH, BMI, and UIC. TSH levels are influenced by these indicators, potentially showing an upward trend with increases in UIC and BMI.

## Data Availability

The raw data supporting the conclusions of this article will be made available by the authors, without undue reservation.
